# The Founder of Plant Taxonomy in China: HU Hsen-Hsu

**DOI:** 10.1007/s13238-021-00877-0

**Published:** 2021-09-26

**Authors:** Xiaojiang Hu, Jinshuang Ma

**Affiliations:** 1 School of Sociology, Beijing Normal University, Beijing, 100875 China; 2 Institute of Botany, Beijing Botanical Garden, Beijing, 100093 China

HU Hsen-Hsu (胡先骕, courtesy name 步曾, Buzeng) was born in Xinjian, Jiangxi Province of China in 1894 (Fig. 1). He received a bachelor’s degree of biology from the University of California in 1916, and a doctorate of applied biology from Harvard University in 1925. From 1918 when he started teaching in college until his death in 1968, in a half-century career Hu made foundational contributions to modern botany in China. He is widely regarded as the founder of Chinese plant taxonomy.

**Figure 1:**
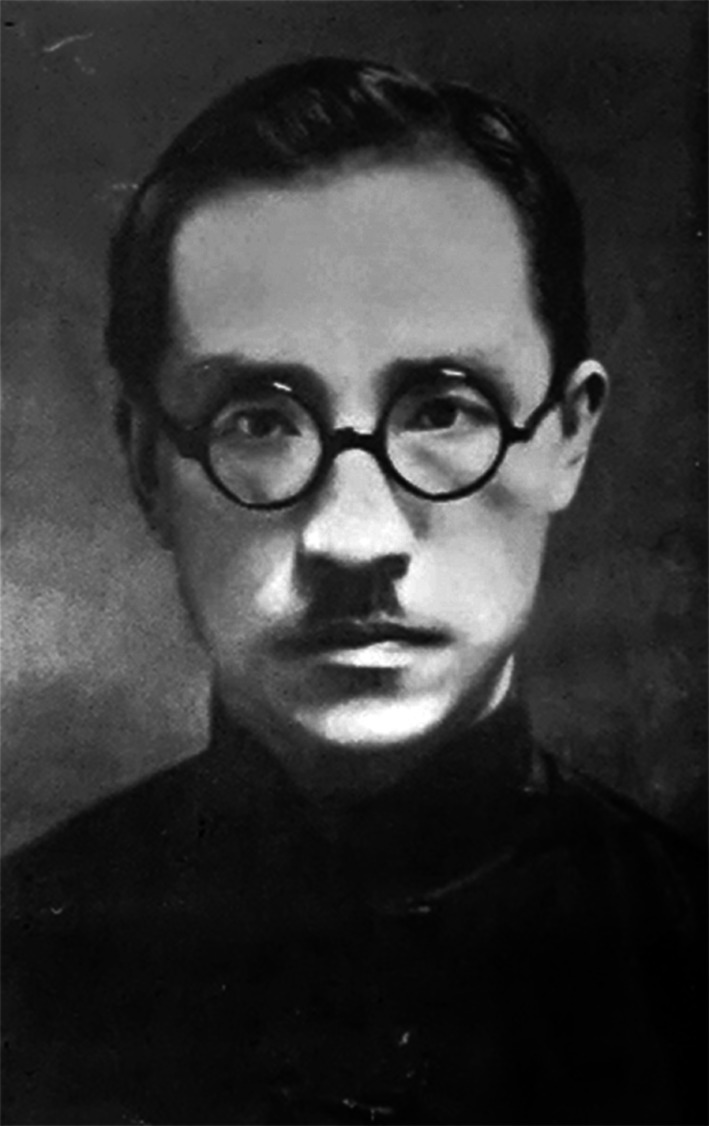
Hu Hsen-Hsu (1940s)

**Figure 2:**
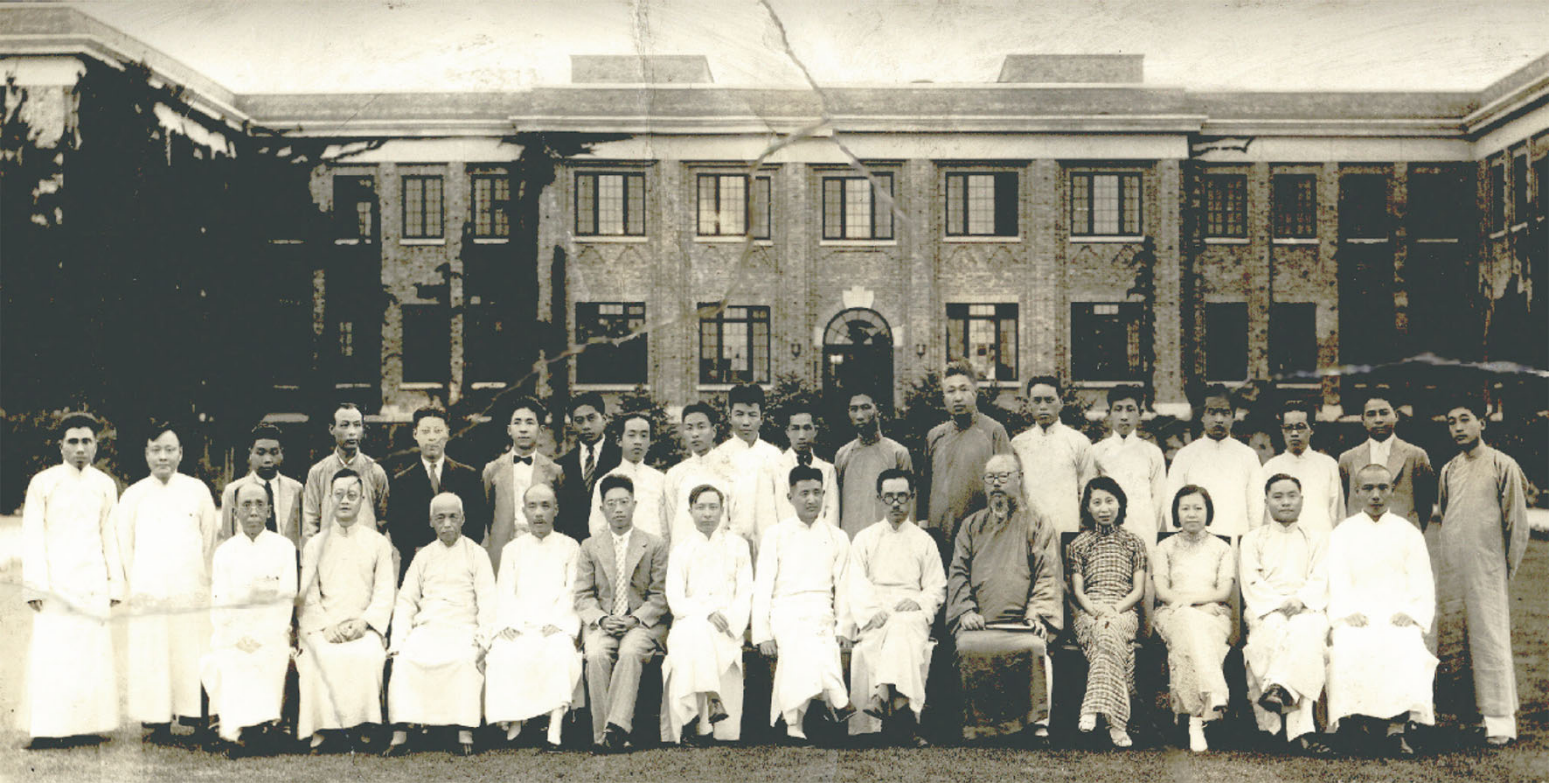
Staff of the Fan Memorial Institute of Biology (in front of the Institute at Peiping, 1936/09/16). Front row: eighth from the left Hu Hsen-Hsu. Back row: sixth from the left Yu Te-Tsun, second from the right Tsai Hse-Tao

## AN ACCOMPLISHED SCIENTIST

As a pioneer scientist, Hu created many “firsts” in Chinese botany. In terms of discovering new plant taxa, Hu was the first Chinese scientist to name a new genus (*Sinojackia*, in 1928) and then a new family (Torricelliaceae, in 1934). Several hundreds of new species of plants were named and described by him. In addition to modern plants, Hu was also a pioneer in paleobotany. *The Miocene Flora of Shandong Province*, *China* (1938, 1940)*,* co-authored with Ralph W. Chaney, was the first work investigating China’s Cenozoic fossil plants, the cornerstone of our knowledge of Asian Cenozoic plants. Hu’s most famous discovery in botany is undoubtedly the “living fossil” *Metasequoia glyptostroboides* (published jointly with CHENG Wan-Chun (郑万钧) in 1948). The discovery of *Metasequoia* became a sensation throughout the world and was hailed as the most important botanic discovery in the 20th century.

In terms of systematic description, Hu’s doctoral dissertation “*Synopsis of Chinese Genera of Phanerogams*” (Harvard University, 1925) was the first monograph that comprehensively surveyed and systematically described phanerogam plants in China. During the following 30 years since its completion, this work was widely used in China as the standard reference work for Chinese plants. In 1950, Hu proposed a new classification system for angiosperm plants. Furthermore in 1965, Hu proposed his own alternative classification system for the entire living world (published in *Taxon*). Both efforts were first of their kind coming from China.

As a prolific scholar, Hu published more than 150 academic papers and more than 20 books throughout his life. But personal achievements alone are not enough to classify a person as a “founder”. The Founder creates a whole disciplinary infrastructure of learning and research, including schools, textbooks, research institutions, academic organizations, academic journals, literature and resource accumulation, and other affine tasks, before the discipline can cultivate talent *en masse* and produce scientific results on a large scale, to the ultimate benefit of society. Hu’s accomplishment in this area is what makes him the seminal figure in the establishment of botany in China.

## A LEADING ENTREPRENEUR IN SCIENCE

Hu’s contribution to the cause of botany in China is vast and comprehensive. With incomparable botanical variety, China is called “the mother of gardens”, and has attracted the curiosity of Western explorers since the 18th century; but until the early 20th century, the true science of botany was still nonexistent in China. After Hu became a professor of the Faculty of Agriculture at Nanjing Higher Teacher’s College in 1918, he immediately planned to collect plant specimens. From 1920 to 1922, Hu personally carried out large-scale plant collections in Zhejiang and Jiangxi provinces. In the following 20 years, Hu continuously organized large-scale plant specimen collections, covering nearly half of China. Among these projects, the Yunnan and Sichuan collection carried out in the 1930s by Tsai Hse-Tao (蔡希陶), Wang Chi-Wu (王启无), Yu Te-Tsun (俞德浚) etc., which resulted in more than 100,000 specimens, was regarded as the landmark of Chinese plant collection. At the same time, Hu sponsored Ching Ren-Chang (秦仁昌) to take high-quality photos of Chinese plant specimens from major herbaria in Europe. The project resulted in 18,337 photos, which meant that “Chinese botanists no longer need to rely on Westerners to identify their own plants!” These new plant specimens and the photos of specimens laid an indispensable physical foundation for the study of Chinese plant taxonomy.

The human capital foundation of botany comes from the systematic organization of educational institutions. In 1921, Hu and zoologist Ping Chi (秉志, 1886–1965) founded the biology department in the National Southeast University. This was the first biology department among Chinese-run universities, apart from missionary universities. Soon in 1922, Hu, Ping and others established China’s first biology research institute—The Biology Laboratory of the Society of Science of China, and its periodical *Contributions from the Biological Laboratory of the Science Society of China*. In 1923, Hu, Chou Ping-Wen (邹秉文) and Chien Chong-Su (钱崇澍) compiled the first college textbook of botany in Chinese “*Advanced Botany*”. In 1928, Hu and Ping founded the Fan Memorial Institute of Biology in Peiping and its periodical the *Bulletin of Fan Memorial Institute of Biology*. In 1933, Hu initiated the Botanical Society of China, and published its periodical *The Chinese Journal of Botany* in the following year. In 1934, Lushan Forestry Botanical Garden was founded in Jiangxi by Hu. And in 1938, he founded the Yunnan Botanical Institute in Kunming.

With this long list of teaching and research institutions, scientific periodicals, and academic organizations, China’s plant taxonomy advanced rapidly from the early 1920s to the late 1930s. In less than 20 years, botany quickly completed its own disciplinary construction and became a modern science in China. It was a dazzling achievement, compared to the pace of many other disciplines. Moreover, Hu built Chinese botany with an international perspective. Most of the scientific periodicals he created were in English or bilingual, and the talent he cultivated were fluent in both Chinese and English. Hu also established extensive partnerships with major botanical institutions around the world. With these efforts, Chinese botany became an equal and important member of the international botanical community.

With his outstanding achievements and leadership, Hu became a widely-respected leader of Chinese botany. He was elected as an academician of the Academia Sinica in 1948. The talent directly cultivated by him was very large. Almost all of the second-generation Chinese plant taxonomists, and about half of the third and fourth-generation Chinese plant taxonomists could be traced back to Hu.

Hu’s life is closely intertwined with the ebbs and flows of Chinese history. His blunt and direct personality enabled him to make great achievements in youth and middle age, but also brought grave adversities to his old age. In addition to science, Hu was outspoken about all social affairs throughout his life, including literature, culture, education and politics. When the “Michurinism” of Lysenko of the USSR dominated China in the 1950s, Hu was the first to openly denounce it as a pseudoscience. This action resulted in his book *Plant Taxonomy Textbook* (1955) being banned, and he himself twice (1955 and 1957) failed to be appointed as an academician of the Chinese Academy of Sciences. Hu died an untimely death in 1968. The conditions of the day eclipsed Hu’s name after his death, and his public position in the history of Chinese botany was dimmed.

But as an old Chinese saying goes, “Thick mountains cannot stop the river from flowing into the sea!” Since the 1990s, interest in his work has been reignited, and much research on Hu has been published. In 2021, 19 volumes of *H. H. Hu*: *Complete Works* (2021) will be published. Hu’s foundational contributions to botany in China will not be buried after all.
